# Exploring the Diversity and Potential Use of Flower-Derived Lactic Acid Bacteria in Plant-Based Fermentation: Insights into Exo-Cellular Polysaccharide Production

**DOI:** 10.3390/foods13182907

**Published:** 2024-09-13

**Authors:** Azadeh Khiabani, Hang Xiao, Anders Peter Wätjen, Miguel Tovar, Vera Kuzina Poulsen, Egon Bech Hansen, Claus Heiner Bang-Berthelsen

**Affiliations:** 1 Research Group for Microbial Biotechnology and Biorefining, National Food Institute, Technical University of Denmark, Kemitrovet, Building 202, 2800 Kongens Lyngby, Denmark; azakhi@food.dtu.dk (A.K.); haxi@food.dtu.dk (H.X.); apewat@food.dtu.dk (A.P.W.); 2 Microbe & Culture Research, Novonesis A/S, Gammel Venlighedsvej 14, 2970 Hørsholm, Denmark; mito@novonesis.com (M.T.); vekp@novonesis.com (V.K.P.); 3 Research Group for Gut, Microbes and Health, National Food Institute, Technical University of Denmark, Kemitrovet, Building 202, 2800 Kongens Lyngby, Denmark; egbh@food.dtu.dk

**Keywords:** exo-cellular polysaccharide, plant-based fermentation, isolation, flower, lactic acid bacteria, texture

## Abstract

Isolation of new plant-derived lactic acid bacteria (LAB) is highly prioritized in developing novel starter cultures for plant-based fermentation. This study explores the diversity of LAB in Danish flowers and their potential use for plant-based food fermentation. A total of 46 flower samples under 34 genera were collected for LAB isolation. By introducing an enrichment step, a total of 61 LAB strains were isolated and identified using MALDI-TOF and 16S rRNA sequencing. These strains represent 24 species across 9 genera, predominantly *Leuconostoc mesenteroides*, *Fructobacillus fructosus*, *Apilactobacillus ozensis*, and *Apilactobacillus kunkeei*. Phenotypic screening for exo-cellular polysaccharide production revealed that 40 strains exhibited sliminess or ropiness on sucrose-containing agar plates. HPLC analysis confirmed that all isolates produced exo-cellular polysaccharides containing glucose, fructose, or galactose as sugar monomers. Therefore, the strains were glucan, fructan, and galactan producers. The suitability of these strains for plant-based fermentation was characterized by using almond, oat, and soy milk. The results showed successful acidification in all three types of plant-based matrices but only observed texture development in soy by *Leuconostoc, Weissella*, *Lactococcus*, *Apilactobacillus*, and *Fructobacillus*. The findings highlight the potential of flower-derived LAB strains for texture development in soy-based dairy alternatives.

## 1. Introduction

Lactic acid bacteria (LAB) are a group of microorganisms widespread in various environments, including plants, terrestrial and marine animals, fermented foods, and the mucosal surfaces of humans. LAB have a long history of safe use in the food industries and are well-known for enhancing food preservation and improving sensory and nutritional qualities [1]. In plant-based fermentation, LAB play a key role in developing the flavor, texture, and shelf life of plant materials. Additionally, they enhance nutrient bioavailability and promote the production of beneficial compounds, such as vitamins and bioactive peptides [2,3].

Traditional starter cultures often face limitations when used for fermenting plant-based materials [3,4]. This has led to a growing interest in isolating novel plant-derived strains for plant-based fermentation. Notably, flowers harbor unique LAB communities distinct from those found in food sources, yet less explored [5]. The unique characteristics of flower-derived LAB may offer solutions to the challenges in plant-based fermentation and hold significant potential for creating novel starter cultures.

In plant-based fermentation, one of the major challenges is to produce products with acceptable taste and texture for customers [3]. Introducing exo-cellular polysaccharide-producing strains offers a promising solution, as exo-cellular polysaccharides contribute to texturization, emulsification, gelling, sweetening, and water-binding capacity properties. These properties have a positive impact on texture, mouthfeel, and reducing syneresis [6]. Exo-cellular polysaccharides potentially provide additional health benefits, including immunomodulatory, prebiotic, anti-inflammatory, anti-biofilm, and antioxidant effects [7,8]. Moreover, in situ production of exo-cellular polysaccharides can enhance food texture while reducing the use of food additives [9,10,11].

It is well known that LAB are capable of synthesizing a variety of exo-cellular polysaccharides, including exopolysaccharides (EPSs) and capsular polysaccharides (CPSs), both of which play a crucial role in texture development within the food industry [12]. CPSs are closely linked to the cell surface, forming the outermost layer of the bacterial cell to protect the cell and mediating direct interactions with the environment. In contrast, EPSs are polysaccharides loosely associated with the cell surface or released into the extracellular medium. They can either be produced extracellularly by enzymes secreted by the bacterium or synthesized intracellularly and then secreted outside the cells, often forming a slime layer [13,14,15].

Exo-cellular polysaccharides are high molecular weight polymeric carbohydrates formed of monosaccharide units linked by glycosidic bonds. Based on their structural composition, exo-cellular polysaccharides can be categorized into homo-polysaccharides and hetero-polysaccharides. Homo-polysaccharides consist of a single type of monosaccharide, such as glucans, fructans, and galactans, and are typically produced with specific enzymes like glucan sucrase or fructan sucrase. Although galactan production has been observed in LAB, the exact genes and enzymes involved remain unclear [16]. On the other hand, hetero-polysaccharides are composed of two or more different sugars, such as pentoses (D-ribose, D-arabinose, and D-xylose), hexoses (D-glucose, D-galactose, and D-mannose), N-acetylated monosaccharides, or uronic acids, and may be modified by non-carbohydrate groups like acetate, pyruvate, sulfate, or succinate [10,13,14]. These polysaccharides are produced at much lower rates due to the complex biosynthesis processes and additional enzymes required for their production.

In this study, we aimed to identify flower-derived LAB and assess their ability to produce exo-cellular polysaccharides. To achieve this, 61 LAB were isolated from a broad variety of flowers in Denmark with a strict de-replication procedure. The production of exo-cellular polysaccharides was characterized both on agar plates and in liquid cultures. Additionally, the monosaccharide composition was also investigated. To evaluate their potential for texturizing plant-based drinks, 25 selected strains were tested in microtiter plates (MTPs) combined with Total Aspirate Dispense Monitoring (TADM) pressure measurements.

## 2. Materials and Methods

### 2.1. Preparation of Flowers

A total of 47 flower samples, representing 34 different species, were collected from various locations in Silkeborg, Aarhus, Odense, and Copenhagen, Denmark, during the summer season. Each flower sample was placed into a sterile 15 mL tube and immediately filled with a sterile 3.5% NaCl solution. After incubating for 5 days at 22 °C, the samples were vortexed, and the supernatants were mixed with 20% glycerol. Then, the samples were stored at −80 °C until further use.

### 2.2. Screening and Isolation of LAB Strain from Flowers

The frozen flower samples were inoculated into MLS-agar supplemented with 1% glucose and 0.5% fructose for LAB screening. The composition of the MLS medium is 5 g/L Meat extract, 2.5 g/L Meat peptone, 5 g/L KH_2_PO_4_, 8 g/L Soy peptone, 10 g/L tryptone, 4 g/L Yeast extract, 5 g/L Sodium acetate, 2 g/L Ammonium citrate, 0.1 g/L ascorbic acid, 0.3 g/L MgSO_4_, 0.1 g/L MnSO_4_, 0.034 g/L FeSO_4_, 1 mL of Tween 80, and 15 g/L Agar. After the pH was adjusted to 6.2, 0.4 g/L Cycloheximide was also added to agar plates to inhibit yeast and fungi growth [17]. For isolation, the plates were made by directly streaking the flower samples supernatant after a brief vortex. After incubating the plates at 30 °C for 48 h, the plates were transferred to the fridge for 24 h to show more distinctive morphology. Then, up to 10 colonies were picked mainly based on their colony morphology, followed by re-streak, and sub-cultured on the MLS-agar at least once for colony purification [17,18].

### 2.3. Identification of Isolated LAB Strains from Flowers Using MALDI-TOF, PCR, and De-Replication

The species identification of the isolated LAB was performed by protein extraction from 24 h grown cultures on MLS-agar plates from a purified single colony using MALDI-TOF Biotype (Bruker Daltonics, Bremen, Germany), which can identify species based on the protein mass to charge (m/z) spectra. The sample preparation process for MALDI-TOF identification is described as follows: Fresh purified cultures from an agar plate are picked using inoculation loops and spread out on spots on a target MALDI-TOF plate. The plate is then treated with 1 µL of 75% ethanol, mixed well, and left to dry. Then 1 µL of 70% formic acid is added to each spot, and the plate is left to dry again. Finally, 1 µL of a saturated matrix is added to each spot. The matrix is prepared by mixing α-cyano-4-hydroxycinnamic acid with 475 µL of water, 500 µL of 100% Acetonitrile, and 25 µL of Trifluoroacetic acid. The plate is left to dry completely before being scanned using a MALDI-TOF Biotype for protein mass spectra detection and identification at the species level by matching with its integrated spectral database library. The identification process will provide a species ID along with a score indicating the similarity between the protein mass spectrum and the database. The instrument gives a score of 1–3, with each score corresponding to the following: score values from 1 to 1.69 are considered not reliable, and thus the genus/species cannot be determined; scores between 1.70 and 1.99 refer to identifications that are only reliable on a genus level; and scores above 2 correspond to identifications that are reliable on a species and genus level [17]. The not-reliable strains (14 strains) with low scores were identified by 16S rRNA sequencing. The fragment (300 bp) was amplified by using a universal primer: following forward (5′ TGGCTCAGGACGAACGCTGGCGGC 3′) and reverse (5′ CCTACTGCTGCCTCCCGTAGGAGT 3′). The PCR procedure is as follows: using PCR master mix (2×) 25 µL, forward primer 1 µM, reverse primer 1 µM, template DNA 10 pg–1 µg, nuclease-free water to 50 µL. Gently vortex the samples and perform PCR using thermal cycling conditions. The PCR program was carried out in a thermal cycler as 5 min of initial denaturation at 95 °C, followed by 35 amplification cycles of denaturation at 95 °C for 30 s, annealing at 58 °C for 30 s, and extension at 72 °C for 40 s. The final elongation was set at 72 °C for 7 min. The PCR products were analyzed via 1% agarose gel electrophoresis, stained with DNA Gold Viewer Dye, and visualized under UV light using a mini gel documentation device (VMR). Then the samples (11 strains) were sent for sequencing. A strict de-replication procedure was then employed to remove duplicate strains from the isolates. If two strains originating from the same sample received the same MALDI-TOF ID with similar scores, only one of the strains will be saved and characterized for further studies [17]. After de-replication, the strains were saved to the DTU National Food Institute Culture Collection (NFICC) with a designated NFICC number.

### 2.4. Screening for Exo-Cellular Polysaccharide Producers

A modified MLS-agar medium supplemented with 1% glucose, 0.5% fructose as a control, and 2% or 6% sucrose was prepared to observe the exo-cellular polysaccharide production abilities of 61 selected strains. Overnight cultures were spotted on modified MLS-agar plates, and the slime formation was monitored following the incubation period of 24, 48, and 72 h at 30 °C, as described previously [19]. Morphologically slimy colonies were further selected for exo-cellular polysaccharide production on modified MLS-agar medium supplemented with 2% sucrose to observe the exo-cellular polysaccharide production abilities of 40 selected strains through visual inspection of the colonies.

### 2.5. Isolation and Purification of Exo-Cellular Polysaccharides

The 40 selected LAB strains were grown in 10 mL of modified MLS-broth medium supplemented with 2% sucrose at 30 °C for 48 h. Isolation of exo-cellular polysaccharides was conducted by following the method as depicted previously [10]. Briefly, following the incubation period, 2 vol of chilled ethanol was added to the culture supernatants obtained following the centrifugation of the bacterial cultures, and the supernatants were left at 4 °C overnight to precipitate the exo-cellular polysaccharides. The exo-cellular polysaccharide pellet was then recovered by centrifugation at 10,000× *g* for 20 min at 4 °C and resuspended with distilled water. This process was repeated twice, using less distilled water each time for the resuspension process.

### 2.6. Determination of Monosaccharides by High-Performance Liquid Chromatography (HPLC) Analysis

The monosaccharide composition is determined by HPLC after treatment for the purified polysaccharides, as previously described [10] with a minor modification: instead of making a 10 mg/mL solution, in this study, the yield EPS from each sample was directly resuspended with 1 mL of distilled water for hydrolysis. An HPLC (ThermoFisher, Boston, MA, USA) analysis is set up in a system equipped with an Aminex HPX-87H (Bio-Rad, Hercules, CA, USA) and a Shodex RI-101 refractive index detector (Showa Denko K.K., Tokyo, Japan). The mobile phase was 5 mm sulfuric acid with a flow rate of 0.5 mL/min. The column oven temperature was maintained at 60 °C. Glucose, galactose, and fructose were used as standard sugars to determine the composition of the purified polysaccharides. Chromatograms for samples and standards were analyzed using Chromeleon 2.0 software (ThermoFisher, Boston, MA, USA).

### 2.7. High-Throughput Screening for Texturing Strains in Plant-Based Drink

The ability of strains to acidify three plant-based drinks was investigated using the color-of-pH method, and their texturing abilities were investigated using TADM, as described in [20]. Here, three types of commercial plant-based drinks were used, and their nutritional content is listed in Table 1. TADM results (pressure versus time curves) were converted into single descriptors (TADM area) by accumulating all the measured pressure points above zero. The pressure was measured every 0.01 s for 3 s. Large TADM areas represented strains resulting in fermented samples with high texture, whereas non-texturing strains were represented by small TADM areas. The strains were considered texturing when giving rise to a TADM area ≥ 800,000 Pa × ms.

## 3. Results and Discussion

### 3.1. Diversity of Plant-Based LAB Strains According to MALDI-TOF, PCR, and De-Replication

**Figure 1:**
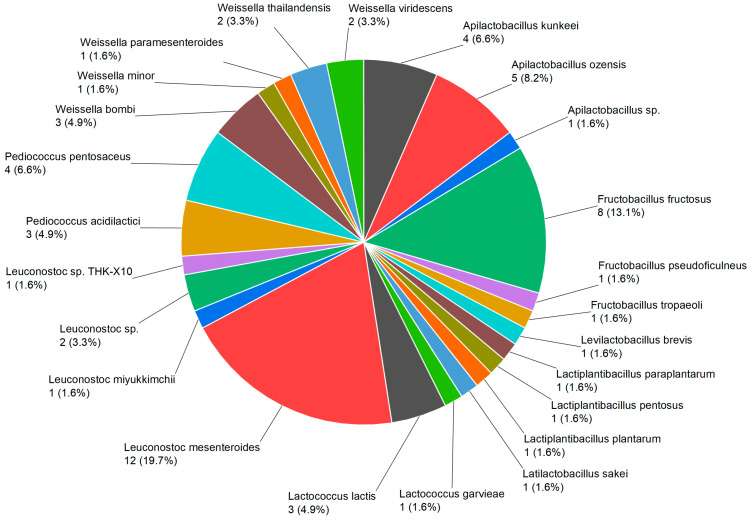
The abundance percentage and diversity of plant-based LAB within the overall LAB community strains isolated from flowers.

To isolate flower-derived LAB, we collected 46 flower samples representing 34 genera from various locations in Denmark during the summer season. By employing MALDI-TOF and 16SrRNA identification (Supplementary File S1), a total of 61 LAB strains belonging to 24 species were isolated after a strict de-replication process (Table 2, Figure 1). The strains belong to nine genera: *Apilactobacillus*, *Fructobacillus*, *Levilactobacillus*, *Lactiplantibacillus*, *Latilactobacillus*, *Lactococcus*, *Leuconostoc*, *Pediococcus*, and *Weissella*. At the species level, the most prevalent LAB species found among all flowers were *Leuconostoc mesenteroides*, with 12 isolated strains. Followed by other frequently occurring species such as *Fructobacillus fructosus* with eight isolates, *Apilactobacillus ozensis* with five isolates, and *Apilactobacillus kunkeei* with four isolates. Our results indicate a wide spread of LAB species on flowers.

In other studies focusing on LAB isolation from flowers, similar occurrences at the species level have been described, which aligns well with the findings from our study [21,22]. In contrast, LAB isolated from fermented vegetables or dairy sources are typically abundant in other species, such as *Lactiplantibacillus plantarum*, *Lacticaseibacillus casei*, *Lacticaseibacillus paracasei*, *Latilactobacillus curvatus*, *Latilactobacillus sakei*, and *Lactobacillus delbrueckii* [17,23,24]. This indicates that flowers harbor a niche for distinct LAB species, with a high abundance of fructophilic LAB. Interestingly, such species are also frequently found inside honeybee guts [25], suggesting a potential microbiota exchange between flowers and pollinators. Unlike LAB derived from dairy and fermented vegetables, flower-derived LAB are less studied. Considering their distinctiveness, it could be a promising resource for investigating novel LAB in food applications.

Although LAB is ubiquitous in nature, isolating and identifying plant-derived LAB strains can be challenging due to their low abundance on plant surfaces and meticulous cultivation conditions [26]. We attempted to use flower-washed water for plating directly but only yielded poor results. To obtain better isolation from flower samples, an enrichment procedure for LAB in each sample needs to be employed before isolation. In this study, inspired by the preparation of fermented vegetables, we introduced a simple method that preserved the flower samples in a 3.5% NaCl solution at room temperature for 5 days. The pre-fermentation resulted in a low pH of around 4 for most of the samples, indicating an enrichment of anaerobes. Additionally, the successful isolation of LAB from different flower samples confirmed the robustness and efficiency of this method.

Regarding the isolation and de-replication process, approximately ten strains per sample were selected based on the appearance of the colonies on agar plates. Therefore, the isolates from each sample only represented the prevalent strains, not the entire LAB community. De-replication is crucial during isolation as it enables high-quality outputs during strain isolation. Furthermore, it allows people to identify new strains without wasting time and resources on strains that have already been discovered.

Overall, the study highlights the potential of flowers as a promising resource for LAB isolation. In comparison to other sources, flowers have received less attention. However, our findings emphasize the distinctness of LAB strains found in flower samples. To develop new starter cultures for plant-based fermentation, it is crucial to study plant-derived LAB, as they likely possess the ability to metabolize plant sugars and proteins, as well as detoxify phenolic compounds found in plant materials [27].

### 3.2. Screening for Polysaccharide-Producing LAB Strains on Different Sucrose-Supplemented Media

**Figure 2:**
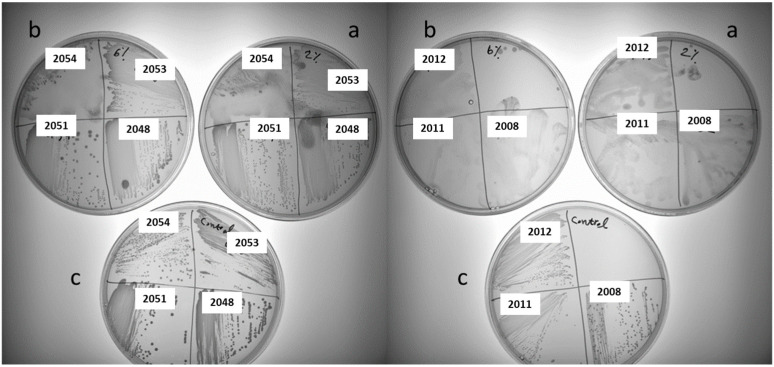
Slime formation associated with homo-EPS production in seven random LAB strains on modified MLS-agar medium supplemented with (a) 2% sucrose, (b) 6% sucrose, and (c) control MLS medium.

Exo-cellular polysaccharides, including exopolysaccharides (EPSs) and capsular polysaccharides (CPSs), are particularly important in the food industry. A practical method to evaluate exo-cellular polysaccharide production is to visually examine the phenotypic traits of the colonies, such as their sliminess or ropiness [10]. The slimy phenotype is recognized by mucilaginous colonies, while the ropy phenotype is identified by the formation of long filaments when an inoculation loop is lifted from the colony surface or cell pellet [28]. As sucrose is usually used for stimulating homo-EPS production in LAB, the screening of homo-EPS production for 61 LAB strains was characterized on MLS plates containing 2% or 6% sucrose. In total, 40 of the tested strains showed varying degrees of sliminess and ropiness, indicating the production of homo-EPS at various levels. More slime was observed when using 2% sucrose compared to using 6% sucrose (Figure 2). No slime or ropy colonies were observed in the control plates with no sucrose added for all tested strains. Hence, we focused on these 40 slime-producing strains for further studies.

**Figure 3:**
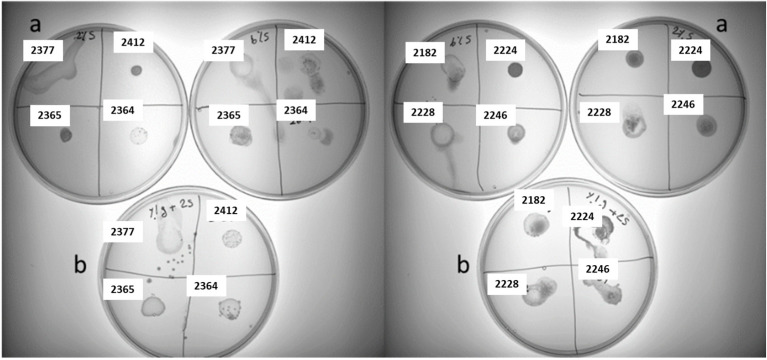
Slime formation associated with homo-EPS production in eight random LAB strains on (a) modified MLS-agar medium supplemented with 2% sucrose and (b) MLS-agar medium supplemented with 2% sucrose and 1% glucose.

Sugar metabolism significantly influences exo-cellular polysaccharide production in LAB. Studies have demonstrated that exo-cellular polysaccharides can be varied in both amount and composition when LAB grows on different sugars [29,30]. Unlike dairy products, which mainly contain lactose as fermentable sugar, the sugar composition in plant-based materials is more complex. Hence, we attempted to investigate exo-cellular polysaccharide production in LAB under mixed-sugar conditions. To simplify the model, exo-cellular polysaccharide production was evaluated in a 2% sucrose plate supplemented with 1% glucose (exemplified in Figure 3). As summarized in Table 3, the results revealed a highly species-dependent pattern in slime production. For example, most *Apilactobacillus ozensis*, *Pediococcus pentosaceus*, *Weissella viridescens*, and *Fructobacillus fructosus* are hampered in slime formation in the presence of glucose. Interestingly, some species exhibited completely opposite behavior, such as *Apilactobacillus kunkeei*, *Leuconostoc miyukkimchii*, *Lactococcus lactis*, *Lactococcus garvieae*, *Leuconostoc mesenteroides*, *Weissella bombi*, and *Weissella minor*. An enhanced slime production was detected in the presence of glucose.

The quantity of exo-cellular polysaccharides synthesized by LAB largely depends on parameters including pH, temperature, oxygen tension, incubation period, metabolic activity, and microbial growth conditions. Nevertheless, the most important factor is the composition of the culture medium and its carbon source [28,31]. Homo-polysaccharide production is induced by adding sucrose [32]. However, in this study, an inhibited slime formation production is observed in some strains when glucose is present. This inhibition may be attributed to carbon catabolite repression, which may inhibit sucrose uptake when glucose is present [33]. In contrast, some strains showed enhanced homo-EPS production in the presence of glucose, which may indicate a difference in the regulation of homo-EPS production [12] or might be a result of higher biomass production. Although the mechanism behind the enhancement or reduction in slime formation in the presence or absence of glucose remains unclear, our findings provide valuable insights when selecting LAB strains for texturizing plant-based materials with different sugar compositions.

### 3.3. Determination of Monosaccharide Composition by HPLC Analysis

**Figure 4:**
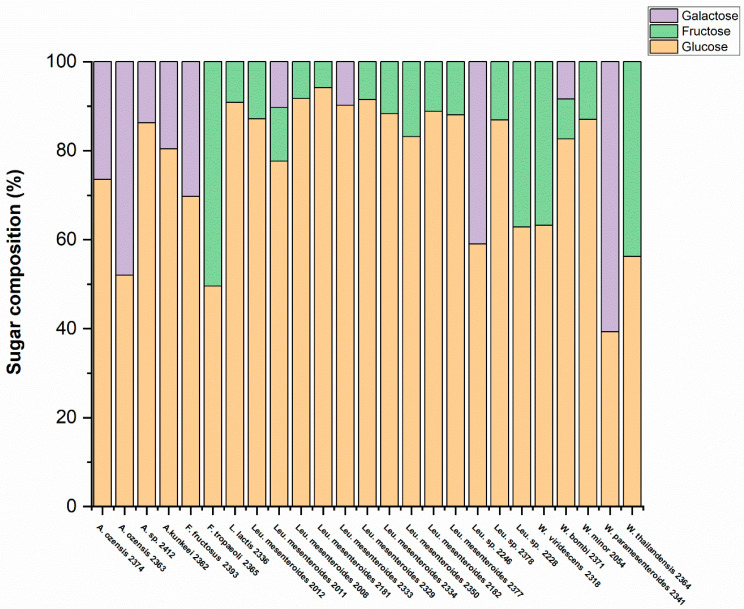
Fingerprint of EPS monosaccharide composition of LAB strains analyzed by HPLC. Glucose is represented by an orange bar color, fructose by a green bar color, and galactose is shown as a purple bar color.

To investigate the monosaccharide composition of exo-cellular polysaccharides, all 40 strains were cultivated in MSL medium supplemented with 2% sucrose. The extraction method used in this study specifically targets EPS, so we focused on analyzing EPS composition. After hydrolysis, 25 strains yielded a satisfactory amount of monosaccharides for compositional analysis (Supplementary File S2). As shown in Figure 4, the sugar composition of EPS produced by each strain is highly dependent on the species level. Specifically, most *Leuconostoc mesenteroides*, *Lactococcus lactis*, and *Weissella minor* produced EPS dominated by glucose, with a small amount of fructose also detected. In contrast, EPS produced from *Fructoacillus tropaeoli*, *Weissella viridescens*, and *Weissella thailandensis* contained both large amounts of glucose and fructose. Furthermore, the EPS produced from *Apilactobacillus kunkeei*, *Apilactobacillus ozensis*, *Apilactobacillus* sp., *Fructobacillus fructosus*, and *Weissella paramesenteroides* consisted of a large amount of glucose and galactose. Overall, all strains had glucose monomers in their composition; some of them had fructose or galactose beside the glucose. Sugar monomers found in *Leuconostoc mesenteroides* (NFICC 2011) and *Weissella bombi* (NFICC 2371) were glucose, fructose, and galactose.

Results of HPLC analysis show that all isolates produced EPS containing glucose, fructose, or galactose as sugar monomers in the EPS structure. This could suggest that these strains produce multiple types of polymers. Previous studies have shown that texturing *Leuconostoc* strains can contain up to five different glucansucrase-like enzymes, potentially leading to the formation of five distinct homo-polysaccharide structures within a single strain. Furthermore, these strains possess gene clusters responsible for the production of hetero-polysaccharides, which may further increase the variety of EPS structures they generate [34]. When comparing the nine genera identified in this study in terms of EPS production efficiency, it was found that *Leuconostoc* had the highest amount of slime production, followed by *Apilactobacillus*, *Fructobacillus*, *Weissella*, *Lactococcus*, *Lactiplantibacillus*, and *Pediococcus*, respectively. According to some studies, a high amount of sugar may contribute to an increase in the production of homo-polysaccharides. Possible explanations for the increased homo-polysaccharide synthesis under the stress of high sugar concentration in some strains include osmosis, the unlimited supply of sugar building blocks, and high energy availability [10,19].

### 3.4. High-Throughput Screening for Texturing Strains

**Figure 5:**
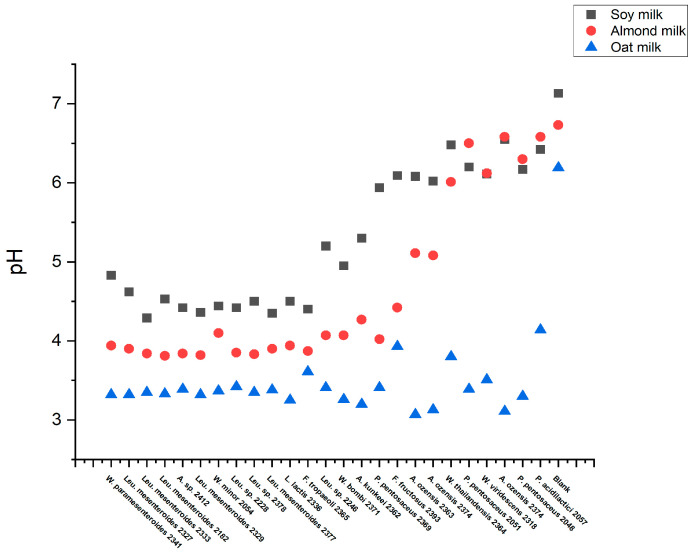
pH values of strains in fermented plant-based drinks.

**Figure 6:**
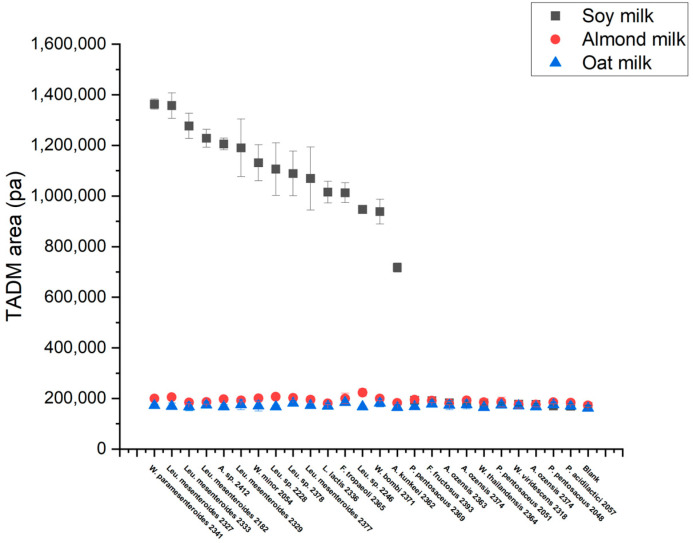
TADM areas of strains in fermented plant-based drinks.

A total of 25 strains selected based on their exo-cellular polysaccharide production observed on the MLS plate were evaluated for their ability to texturize oat, almond, and soy drinks without added sugars (Table 1). The fermentation was carried out using 96-deep-well plates at 30 °C for 1 day. The starting pH was about 7. At the end of fermentation, the endpoint pH and texture in each sample were investigated. An endpoint pH below 5.5 was considered indicative of acidification, while a TADM area exceeding 800,000 Pa × ms was regarded as texturing. The results showed that all strains could acidify oat but were not texturing. In contrast, when using soy and almond drinks, several strains exhibited a similar acidification tendency in both substrates depending on the strain used (Figure 5). Interestingly, texturization was observed only in the soy drink, likely due to its relatively high protein content compared to oat and almond drinks. A total of 15 strains resulted in an enhanced texture in soy, including 7 *Leuconostoc mesenteroides*, 1 *Leuconostoc* sp., 1 *Weissella cibaria*, 1 *Weissella minor*, 1 *Weissella paramesenteroides*, 1 *Apilactobacillus kunkeei*, 1 *Apilactobacillus* sp., 1 *Fructobacillus tropaeoli*, and 1 *Lactococcus lactis*. As expected, texture was found to be highly dependent on acidification. In acidified samples, TADM areas varied from 800,000 to 1,400,000 Pa × ms, indicating significant differences in texturing ability among strains (Figure 6).

The selection of LAB strains suitable for plant-based fermentation is instrumental in enhancing the texture and mouthfeel of plant-based products. The unique textural properties of dairy products are largely due to their protein and fat content, which are different in plant-based matrices. LAB have the potential to address these challenges by producing exo-cellular polysaccharides during fermentation, which can improve the texture of plant-based products. For instance, in plant-based yogurt alternatives, LAB can contribute to a creamier texture and more desirable mouthfeel, making these products more appealing to consumers [35]. The acidification process driven by LAB also plays a crucial role in modifying the structure of plant-based proteins. This can lead to the formation of a gel-like consistency, similar to that found in traditional yogurt and cheese, which is particularly valuable in creating plant-based versions of these products [36]. This could explain why texturing was only observed in soy drinks in this study since its protein content is around four times higher than that in almond and oat drinks. In oat and almond milk, elevated TADM aspiration pressures were also detected from all strains after fermentation compared to the blank samples. This could be due to the produced exo-cellular polysaccharides being insufficient for yogurt-like texture formation.

Exo-cellular polysaccharide production in LAB has been well studied; however, most industrial strains are tailored for making yogurt and cheese. Our study showed that several traditional dairy starters, such as *Leuconostoc*, *Weissella*, and *Lactococcus*, enabled texture formation in soy as well. It is worth noting that *Leuconostoc* normally grows poorly in milk alone and is often cultured with other LAB like *Lactococcus lactis* in dairy fermentation [37]. In this study, eight *Leuconostoc* strains showed better texture development in soy milk, demonstrating their promising role shift from the dairy to the plant-based section. In addition, *Apilactobacillus* sp. and *Fructobacillus tropaeoli* also showed better texture development in soy milk. These LAB species are rarely studied for EPS production and plant-based fermentation. Our results highlight the potential of broadening the LAB diversity used for plant-based fermentation. Furthermore, the plant-based drinks used in this study are unsweetened. The ability of diverse strains to acidify and texturize plant-based matrices based on their natural nutritional composition highlights their robustness and flexibility in developing plant-based fermented products.

Different types of cow milk have a similar profile of proteins, fats, and sugars. In contrast, plant-based drinks vary significantly in nutrient compositions and physicochemical properties. These differences can have a substantial impact on the microbial fermentation process in different plant-based matrices [17]. To fully explore the potential of LAB in plant-based fermentations, it is essential to know how different plant matrices interact with LAB. Factors such as protein, fat, and carbohydrate availability need to be considered when designing new products. By fine-tuning fermentation conditions and selecting strains with specific properties, it is possible to optimize the textural and sensory attributes of plant-based products.

## 4. Conclusions

This study demonstrates a wide distribution of LAB in Danish flowers, along with an innovative and efficient method for isolating LAB from flower samples. A total of 61 LAB strains were isolated and identified by using MALDI-TOF and 16S rRNA sequencing from 46 flower samples representing 34 genera. Additionally, we highlight the potential use of flower-derived LAB for plant-based food fermentation. The robust acidification and texture development observed, particularly in soy milk by *Leuconostoc*, *Weissella*, *Lactococcus*, *Apilactobacillus*, and *Fructobacillus*, indicate their significant role in enhancing the quality and diversity of plant-based fermented products. Further genomic studies of the strains and compositional studies for different plant-based materials are essential to optimize their application in plant-based food fermentation. By understanding the interaction between LAB and different plant-based matrices, we can unlock new possibilities for creating novel, high-quality plant-based products that meet the demands of today’s consumers.

## Figures and Tables

**Table 1:** Nutrient content of used plant-based drink.

	Oat, Isola Bio	Almond, Ecomil	Soy, Naturli
Energy	215 kJ/51 kcal	134.00 kJ/32.00 kcal	153 kJ/37 kcal
Fat	1.0 g	2.10 g	2.1 g
Carbohydrate	9.0 g	3.30 g	0.6 g
-Here sugar	4.2 g	<0.30 g	0.6 g
Protein	1.0 g	1.00 g	3.7 g
Salt	0.08 g	0.14 g	0.09 g

**Table 2:** Overview of the diversity of plant-based LAB strains isolated from flowers.

No.	Species	NFICC ID	City	Flower
1	*Apilactobacillus kunkeei*	2373	Odense	Verbascum
2	*Apilactobacillus kunkeei*	2324	Copenhagen	Rosa
3	*Apilactobacillus kunkeei*	2359	Aarhus	Fallopia
4	*Apilactobacillus kunkeei*	2362	Silkeborg	Rubus
5	*Apilactobacillus ozensis*	2370	Odense	Potentilla
6	*Apilactobacillus ozensis*	2374	Odense	Jacobaea
7	*Apilactobacillus ozensis*	2363	Silkeborg	Rubus
8	*Apilactobacillus ozensis*	2366	Silkeborg	Lavandula
9	*Apilactobacillus ozensis*	2368	Odense	Jacobaea
10	*Apilactobacillus* sp.	2412	Silkeborg	Lavandula
11	*Fructobacillus fructosus*	2323	Copenhagen	Rosa
12	*Fructobacillus fructosus*	2321	Copenhagen	Geranium
13	*Fructobacillus fructosus*	2361	Silkeborg	Rubus
14	*Fructobacillus fructosus*	2347	Copenhagen	Rosa
15	*Fructobacillus fructosus*	2325	Copenhagen	Rubus
16	*Fructobacillus fructosus*	2319	Copenhagen	Syringa
17	*Fructobacillus fructosus*	2376	Odense	Convolvulus
18	*Fructobacillus fructosus*	2393	Silkeborg	Lavandula
19	*Fructobacillus pseudoficulneus*	2222	Aarhus	Betonica
20	*Fructobacillus tropaeoli*	2365	Silkeborg	Lavandula
21	*Levilactobacillus brevis*	2055	Aarhus	Eschscholzia
22	*Lactiplantibacillus paraplantarum*	2184	Odense	Cirsium
23	*Lactiplantibacillus pentosus*	2185	Odense	Cirsium
24	*Lactiplantibacillus plantarum*	2183	Odense	Cirsium
25	*Latilactobacillus sakei*	2317	Copenhagen	Stellaria
26	*Lactococcus garvieae*	2331	Copenhagen	Hyacinthus
27	*Lactococcus lactis*	2358	Aarhus	Erythranthe
28	*Lactococcus lactis*	2332	Copenhagen	Hyacinthus
29	*Lactococcus lactis*	2336	Copenhagen	Trifolium
30	*Leuconostoc mesenteroides*	2012	Copenhagen	Forsythia
31	*Leuconostoc mesenteroides*	2011	Copenhagen	Prunus
32	*Leuconostoc mesenteroides*	2008	Copenhagen	Aubrieta
33	*Leuconostoc mesenteroides*	2181	Aarhus	Telekia
34	*Leuconostoc mesenteroides*	2333	Copenhagen	Prunus
35	*Leuconostoc mesenteroides*	2343	Copenhagen	Trifolium
36	*Leuconostoc mesenteroides*	2329	Copenhagen	Ranunculus
37	*Leuconostoc mesenteroides*	2327	Copenhagen	Hottonia
38	*Leuconostoc mesenteroides*	2334	Copenhagen	Trifolium
39	*Leuconostoc mesenteroides*	2350	Copenhagen	Bellis
40	*Leuconostoc mesenteroides*	2182	Aarhus	Telekia
41	*Leuconostoc mesenteroides*	2377	Aarhus	Campanula
42	*Leuconostoc miyukkimchii*	2224	Copenhagen	Rosa
43	*Leuconostoc* sp.	2228	Aarhus	Dasiphora
44	*Leuconostoc* sp.	2378	Aarhus	Black mullein
45	*Leuconostoc* sp. *THK-X10*	2246	Copenhagen	Rosa
46	*Pediococcus acidilactici*	2053	Aarhus	Betonica
47	*Pediococcus acidilactici*	2057	Aarhus	Fuchsia
48	*Pediococcus acidilactici*	2357	Aarhus	Erythranthe
49	*Pediococcus pentosaceus*	2051	Aarhus	Hydrangea
50	*Pediococcus pentosaceus*	2048	Aarhus	Dasiphora
51	*Pediococcus pentosaceus*	2379	Aarhus	Teucrium
52	*Pediococcus pentosaceus*	2369	Odense	Artemisia
53	*Weissella bombi*	2356	Aarhus	Erythranthe
54	*Weissella bombi*	2346	Aarhus	Teucrium
55	*Weissella bombi*	2371	Odense	Agastache
56	*Weissella minor*	2054	Aarhus	Betonica
57	*Weissella paramesenteroides*	2341	Copenhagen	Trifolium
58	*Weissella thailandensis*	2056	Odense	Convolvulus
59	*Weissella thailandensis*	2364	Silkeborg	Lavandula
60	*Weissella viridescens*	2320	Copenhagen	Cardamine
61	*Weissella viridescens*	2318	Copenhagen	Cotoneaster

**Table 3:** Evaluation of homo-EPS production of the LAB strains on modified MLS-agar medium supplemented with 2% sucrose or 2% sucrose and 1% glucose.

NFICC Codes	Species	2% Sucrose		2% Sucrose and 1% Glucose	
		Slimy	Ropy	Slimy	Ropy
2324	*Apilactobacillus kunkeei*	−	+	+	+
2362	*Apilactobacillus kunkeei*	+	+	++	−
2370	*Apilactobacillus ozensis*	++	+	−	+
2374	*Apilactobacillus ozensis*	+++	−	++	−
2363	*Apilactobacillus ozensis*	−	+	−	+
2366	*Apilactobacillus ozensis*	−	+++	−	++
2347	*Fructobacillus fructosus*	+	−	−	+
2185	*Lactobacillus pentosus*	+−	−	−	−
2183	*Lactiplantibacillus plantarum*	+−	−	−	−
2331	*Lactococcus garvieae*	+−	+	+−	+
2336	*Lactococcus lactis*	+	++	++	+
2012	*Leuconostoc mesenteroides*	−	+	++	+−
2011	*Leuconostoc mesenteroides*	+−	+−	+−	+
2008	*Leuconostoc mesenteroides*	−	+	+	+
2181	*Leuconostoc mesenteroides*	+−	−	++	−
2333	*Leuconostoc mesenteroides*	+	+	++	+
2329	*Leuconostoc mesenteroides*	+	−	+	−
2327	*Leuconostoc mesenteroides*	+	−	+	−
2334	*Leuconostoc mesenteroides*	+−	−	+	−
2350	*Leuconostoc mesenteroides*	−	+−	−	−
2182	*Leuconostoc mesenteroides*	+	−	++	−
2224	*Leuconostoc miyukkimchii*	−	+	+	++
2228	*Leuconostoc* sp.	+	+	+	+
2246	*Leuconostoc* sp. *THK-X10*	+	−	+	+
2057	*Pediococcus acidilactici*	−	+++	−	+++
2051	*Pediococcus pentosaceus*	+	−	−	−
2048	*Pediococcus pentosaceus*	+	+	−	−
2369	*Pediococcus pentosaceus*	−	+++	−	+++
2379	*Pediococcus pentosaceus*	−	+	−	+
2371	*Weissella bombi*	++	−	++	−
2054	*Weissella minor*	+	+	++	+
2341	*Weissella paramesenteroides*	+	+	+	+
2056	*Weissella thailandensis*	+	−	++	−
2318	*Weissella viridescens*	+	−	−	+−
2393	*Fructobacillus fructosus*	+	−	−	−
2378	*Leuconostoc* sp.	+	−	++	+
2377	*Leuconostoc mesenteroides*	+	−	+	+
2412	*Apilactobacillus* sp.	+	−	−	−
2365	*Fructobacillus tropaeoli*	+	−	−	−
2364	*Weissella thailandensis*	+	−	−	++

## Data Availability

The original contributions presented in the study are included in the article and Supplementary Materials; further inquiries can be directed to the corresponding author.

## Supplementary Materials

The following supporting information can be downloaded at https://www.mdpi.com/article/10.3390/foods13182907/s1, Supplementary File S1. Raw data file for MALDI-TOF and 16srRNA identification; Supplementary File S2. HPLC test for monosaccharide composition.

## References

[1] Yang X., Hong J., Wang L., Cai C., Mo H., Wang J., Fang X., Liao Z. (2024). Effect of Lactic Acid Bacteria Fermentation on Plant-Based Products. Fermentation.

[2] Wang Y., Tuccillo F., Lampi A.M., Knaapila A., Pulkkinen M., Kariluoto S., Coda R., Edelmann M., Jouppila K., Sandell M. (2022). Flavor challenges in extruded plant-based meat alternatives: A review. Compr. Rev. Food Sci. Food Saf..

[3] Montemurro M., Pontonio E., Coda R., Rizzello C.G. (2021). Plant-based alternatives to yogurt: State-of-the-art and perspectives of new biotechnological challenges. Foods.

[4] Pua A., Tang V.C.Y., Goh R.M.V., Sun J., Lassabliere B., Liu S.Q. (2022). Ingredients, processing, and fermentation: Addressing the organoleptic boundaries of plant-based dairy analogues. Foods.

[5] Endo A., Maeno S., Tanizawa Y., Kneifel W., Arita M., Dicks L., Salminen S. (2018). Fructophilic lactic acid bacteria, a unique group of fructose-fermenting microbes. Appl. Environ. Microbiol..

[6] Huang W., Dong A., Pham H.T., Zhou C., Huo Z., Wätjen A.P., Prakash S., Bang-Berthelsen C.H., Turner M.S. (2023). Evaluation of the fermentation potential of lactic acid bacteria isolated from herbs, fruits and vegetables as starter cultures in nut-based milk alternatives. Food Microbiol..

[7] Wu J., Han X., Ye M., Li Y., Wang X., Zhong Q. (2023). Exopolysaccharides synthesized by lactic acid bacteria: Biosynthesis pathway, structure-function relationship, structural modification and applicability. Crit. Rev. Food Sci. Nutr..

[8] Özpınar F.B., İspirli H., Kayacan S., Korkmaz K., Dere S., Sagdic O., Alkay Z., Tunçil Y.E., Ayyash M., Dertli E. (2024). Physicochemical and structural characterisation of a branched dextran type exopolysaccharide (EPS) from Weissella confusa S6 isolated from fermented sausage (Sucuk). Int. J. Biol. Macromol..

[9] Li C., Li W., Chen X., Feng M., Rui X., Jiang M., Dong M. (2014). Microbiological, physicochemical and rheological properties of fermented soymilk produced with exopolysaccharide (EPS) producing lactic acid bacteria strains. LWT-Food Sci. Technol..

[10] Yalmanci D., İspirli H., Dertli E. (2022). Identification of Lactic Acid Bacteria (LAB) from pre-fermented liquids of selected cereals and legumes and characterization of their exopolysaccharides (EPS). Food Biosci..

[11] Poulsen V.K., Koza A., Al-Nakeeb K., Oeregaard G. (2020). Screening for texturing Leuconostoc and genomics behind polysaccharide production. FEMS Microbiol. Lett..

[12] Zeidan A.A., Poulsen V.K., Janzen T., Buldo P., Derkx P.M., Øregaard G., Neves A.R. (2017). Polysaccharide production by lactic acid bacteria: From genes to industrial applications. FEMS Microbiol. Rev..

[13] Daba G.M., Elnahas M.O., Elkhateeb W.A. (2021). Contributions of exopolysaccharides from lactic acid bacteria as biotechnological tools in food, pharmaceutical, and medical applications. Int. J. Biol. Macromol..

[14] Jurášková D., Ribeiro S.C., Silva C.C. (2022). Exopolysaccharides produced by lactic acid bacteria: From biosynthesis to health-promoting properties. Foods.

[15] Lynch K.M., Coffey A., Arendt E.K. (2018). Exopolysaccharide producing lactic acid bacteria: Their techno-functional role and potential application in gluten-free bread products. Food Res. Int..

[16] Schmid J., Sieber V., Rehm B. (2015). Bacterial exopolysaccharides: Biosynthesis pathways and engineering strategies. Front. Microbiol..

[17] Kavitake D., Devi P.B., Shetty P.H. (2020). Overview of exopolysaccharides produced by Weissella genus—A review. Int. J. Biol. Macromol..

[18] Xiao H., Molina G.E.S., Tovar M., Quoc H.M., Hansen E.B., Bang-Berthelsen C.H. (2023). Isolation and characterization of plant-based lactic acid bacteria from spontaneously fermented foods using a new modified medium. LWT.

[19] Molina G.E.S., Shetty R., Xiao H., Wätjen A.P., Tovar M., Bang-Berthelsen C.H. (2022). Development of a novel lactic acid bacteria starter culture approach: From insect microbiome to plant-based fermentations. LWT.

[20] Iosca G., De Vero L., Di Rocco G., Perrone G., Gullo M., Pulvirenti A. (2022). Anti-spoilage activity and exopolysaccharides production by selected lactic acid bacteria. Foods.

[21] Poulsen V.K., Derkx P., Oregaard G. (2019). High-throughput screening for texturing *Lactococcus* strains. FEMS Microbiol. Lett..

[22] Ruiz Rodríguez L.G., Mohamed F., Bleckwedel J., Medina R., De Vuyst L., Hebert E.M., Mozzi F. (2019). Diversity and functional properties of lactic acid bacteria isolated from wild fruits and flowers present in Northern Argentina. Front. Microbiol..

[23] Saleh G. (2020). Isolation and characterization of unique fructophilic Lactic acid bacteria from different flower sources. Iraqi J. Agric. Sci..

[24] Anacarso I., Bassoli L., Sabia C., Iseppi R., Condò C. (2015). Isolation and identification of lactic acid bacteria from plants and other vegetable matrices and microbial recombination with *Enterococcus* spp.. Am. Res. Thoughts.

[25] Terzić-Vidojević A., Veljović K., Tolinački M., Živković M., Lukić J., Lozo J., Fira Đ., Jovčić B., Strahinić I., Begović J. (2020). Diversity of non-starter lactic acid bacteria in autochthonous dairy products from Western Balkan Countries-technological and probiotic properties. Food Res. Int..

[26] Iorizzo M., Pannella G., Lombardi S.J., Ganassi S., Testa B., Succi M., Sorrentino E., Petrarca S., De Cristofaro A., Coppola R. (2020). Inter-and intra-species diversity of lactic acid bacteria in Apis mellifera ligustica colonies. Microorganisms.

[27] Aleklett K., Hart M., Shade A. (2014). The microbial ecology of flowers: An emerging frontier in phyllosphere research. Botany.

[28] Pimentel T.C., de Oliveira L.I.G., Macedo E.d.L.C., Costa G.N., Dias D.R., Schwan R.F., Magnani M. (2021). Understanding the potential of fruits, flowers, and ethnic beverages as valuable sources of techno-functional and probiotics strains: Current scenario and main challenges. Trends Food Sci. Technol..

[29] Ruas-Madiedo P., Salazar N., de los Reyes-Gavilán C.G. (2010). Exopolysaccharides produced by lactic acid bacteria in food and probiotic applications. Microbial Glycobiology.

[30] Fuso A., Bancalari E., Castellone V., Caligiani A., Gatti M., Bottari B. (2023). Feeding lactic acid bacteria with different sugars: Effect on exopolysaccharides (EPS) production and their molecular characteristics. Foods.

[31] Paulo E.M., Vasconcelos M.P., Oliveira I.S., de Jesus Affe H.M., Nascimento R., de Melo I.S., de Abreu Roque M.R., de Assis S.A. (2012). Método alternativo de triagem de bactérias láticas produtoras de exopolissacarídeos com confirmação rápida. Food Sci. Technol..

[32] Subramanian S.B., Yan S., Tyagi R.D., Surampalli R. (2010). Extracellular polymeric substances (EPS) producing bacterial strains of municipal wastewater sludge: Isolation, molecular identification, EPS characterization and performance for sludge settling and dewatering. Water Res..

[33] van Hijum S.A., Kralj S., Ozimek L.K., Dijkhuizen L., van Geel-Schutten I.G. (2006). Structure-function relationships of glucansucrase and fructansucrase enzymes from lactic acid bacteria. Microbiol. Mol. Biol. Rev..

[34] Görke B., Stülke J. (2008). Carbon catabolite repression in bacteria: Many ways to make the most out of nutrients. Nat. Rev. Microbiol..

[35] Korcz E., Varga L. (2021). Exopolysaccharides from lactic acid bacteria: Techno-functional application in the food industry. Trends Food Sci. Technol..

[36] Poulsen V.K., Moghadam E.G., Kračun S.K., Svendsen B.A., Nielsen W.M., Oregaard G., Krarup A. (2022). Versatile Lactococcus lactis strains improve texture in both fermented milk and soybean matrices. FEMS Microbiol. Lett..

[37] Erkus O., De Jager V.C., Spus M., van Alen-Boerrigter I.J., Van Rijswijck I.M., Hazelwood L., Janssen P.W., Van Hijum S.A., Kleerebezem M., Smid E.J. (2013). Multifactorial diversity sustains microbial community stability. ISME J..

