# Supplementary material for: Exploring the Diversity and Potential Use of Flower-Derived Lactic Acid Bacteria in Plant-Based Fermentation: Insights into Exo-Cellular Polysaccharide Production
Source: Foods. 2024 Sep 13;13(18):2907. doi: 10.3390/foods13182907 (PMC11430985; doi:10.3390/foods13182907)
Supplement: Supplementary file 1 [file foods-13-02907-s001.zip › Supplementary File S2.pdf]

HPLC quantification for monosaccharide composition

Strain	Sugar concentration (g/L)* Glucose	Fructose	Galactose
A. sp. 2412	4.30	0.00	0.68
F. fructosus 2393	1.88	0.00	0.82
P.pentosaceus 2379	0.00	0.00	0.45
Leu. sp. 2378	10.76	1.62	0.00
Leu. mesenteroides 2377	8.42	1.14	0.00
A. ozensis 2374	1.84	0.00	0.66
W. bombi 2371	6.57	0.72	0.67
A. ozensis 2370	0.00	0.00	0.25
P. pentosaceus 2369	0.00	0.00	0.35
A. ozensis 2366	0.00	0.00	0.39
F. tropaeoli 2365	1.04	1.06	0.00
W. thailandensis 2364	1.73	1.35	0.00
A. ozensis 2363	0.38	0.00	0.35
A.kunkeei 2362	2.45	0.00	0.59
Leu. mesenteroides 2350	17.12	3.46	0.00
F. fructosus 2347	0.00	0.00	0.06
W. paramesenteroides 2341	0.21	0.00	0.33
L. lactis 2336	29.00	2.91	0.00
Leu. mesenteroides 2334	3.22	0.42	0.00
Leu. mesenteroides 2333	4.87	0.00	0.53
L. garvieae 2331	0.00	0.00	0.44
Leu. mesenteroides 2329	21.95	2.05	0.00
Leu.mesenteroides 2327	0.00	0.00	0.49
A. kunkeei 2324	0.00	0.00	0.12
W. viridescens 2318	3.77	2.19	0.00
Leu. sp. 2246	0.16	0.00	0.11
Leu. sp. 2228	5.41	3.20	0.00
Leu. miyukkimchii 2224	0.00	0.00	0.50
L. pentosus 2185	0.00	0.00	0.26
L. plantarum 2183	0.00	0.00	0.28
Leu. mesenteroides 2182	18.63	2.33	0.00
Leu. mesenteroides 2181	13.20	0.81	0.00
P. acidilactici 2057	0.01	0.00	0.42
W.thailandensis 2056	0.03	0.00	0.42
W. minor 2054	13.66	2.03	0.00
P.pentosaceus 2051	0.00	0.00	0.52
P.pentosaceus 2048	0.00	0.00	0.45
Leu. mesenteroides 2012	10.87	1.60	0.00
Leu. mesenteroides 2011	4.36	0.67	0.58
Leu. mesenteroides 2008	7.96	0.72	0.00

* The values represent the sugar concentration directly from the HPLC samples, with 20 µl injection volume; they do not represent the EPS amount from the original culture broth. All samples were obtained from the same processing procedure.
